# Medical hypothesis: can gonadotropins influence thyroid volume in women with PCOS?

**DOI:** 10.1186/1756-6614-5-17

**Published:** 2012-11-29

**Authors:** Evrim Cakir, Mustafa Sahin, Erman Cakal, Mustafa Ozbek, Tuncay Delibasi

**Affiliations:** 1 Department of Endocrinology and Metabolic Diseases, Amasya Sabuncuoglu Serefettin Hospital, Amasya, Turkey; 2 Department of Endocrinology and Metabolic Diseases, Ankara University Hospital, Ankara, Turkey; 3 Department of Endocrinology and Metabolic Diseases, Diskapi Yildirim Beyazit Training and Research Hospital, Ankara, Turkey

**Keywords:** Thyroid volume, Luteinizing hormone, Polycystic ovary syndrome

## Abstract

It has been reported that luteinizing hormone (LH) had thyropropic effect on rat and human thyroid membrane. It has been known that patients with PCOS have elevated LH levels in comparison to healthy controls.

The goiter prevalence is more common in women than in men regardless of population. The higher incidence of thyroid diseases in women has been previously attributed to higher estradiol levels. Estradiol has been shown to enhance proliferative and mitogenic activities of thyroid cells. However, in recent years chronic estradiol treatment has been shown to reduce volume densities of thyroid follicles, follicular epithelium and thyroid gland volume. It is thought to be due to LH suppression.

Therefore we suggested that increased LH levels might provide a stimulus for growth on thyroid and alter thyroid function. Therefore patients with PCOS who had elevated LH levels should be treated by combined estradiol pills such as estrogen-progestin contraceptives for suppression of LH secretion. Further studies are needed to evaluate the association between LH, LH suppression and thyroid volume in patients with PCOS.

## Background

Human chorionic gonadotropin (hCG) secreting placental tumors such as hydatidiform moles and choriocarcinomas have been found to be associated with hyperthyroidism [1] and HCG has been reported to increase TSH receptor expression, thyroid hormone secretion, iodide uptake, organification, adenylate cyclase, and deoxribonucleic acid synthesis in rat and also cultured human thyrocytes [2-4].

Luteinizing hormone is a glycoprotein hormone like TSH and has similar alpha subunit. In Carayon et al. study LH has been identified to increase thyroid adenylate cylase activity 65 times more potently than HCG in human thyroid membranes [5]. Also, in Yoshimura et al. study human LH has been found to be more potent than HCG in binding to the TSH receptor and stimulating adenylate cyclase [6]. As mentioned above, hCG and LH have thyrotropic effect on rat and human thyroid membrane.

Our hypotheses is increased LH levels might have a growth effect on thyroid gland in patients with polycystic ovary syndrome (PCOS). Polycystic ovary syndrome is a common endocrine disorder affecting at least five to 10% of women of reproductive age [7].

The goiter prevalence is more common in women than in men regardless of population [8]. However, there have been no data on increased thyroid volume or goitre prevalence in women with PCOS versus women with regular menstrual cycles. The higher incidence of thyroid diseases in women has been previously attributed to higher estradiol levels. Estradiol has been shown to enhance proliferative and mitogenic activities of thyroid cells [9,10]. However, in recent years chronic estradiol treatment has been shown to reduce volume densities of thyroid follicles, follicular epithelium and thyroid gland volume [11,12]. Despite the enhancing effect of estradiol on thyroid cells, the reducing effect of chronic estradiol treatment on thyroid volume can be related to LH suppression.

It has been known that patients with PCOS have elevated LH levels [13,14] and also have higher gonadotrophin-releasing hormone (GnRH)-stimulated LH concentrations in comparison to healthy controls [15]. In relation to present hypotheses in Knudsen et al. study 3712 women were evaluated and found that oral contraceptives were related with a lower thyroid volume and reduced risk of goiter. In this study the oral contraceptives users had goitre four times less common than non-users [12]. In this case, the LH suppression with oral contraceptives including estradiol might be the factor for decreased thyroid volumes consistent with present hypotheses. However, this literature applies to general population of women, and not to women with PCOS and additionally, in literature search there is no data that the use oral contraceptives in women with PCOS is associated with reduction of thyroid volume or lower goitre prevalence.

In the recent hypotheses increased LH levels might provide a stimulus for growth on thyroid and alter thyroid function. Therefore patients with PCOS who had elevated LH levels should be treated by combined estradiol pills such as estrogen-progestin contraceptives for suppression of LH secretion. Further studies are needed to evaluate the association between LH, LH suppression and thyroid volume in patients with PCOS.

## References

[B1] Yoshimura M Hershman JM Thyrotropic action of human chorionic gonadotropin Thyroid 1995 5 5 425 434 10.1089/thy.1995.5.425 8563483

[B2] Hershman JM Role of human chorionic gonadotropin as a thyroid stimulator J Clin Endocrinol Metab 1992 74 2 258 259 10.1210/jc.74.2.258 1730804

[B3] Hershman JM Lee HY Sugawara M Mirell CJ Pang XP Yanagisawa M Pekary AE Human chorionic gonadotropin stimulates iodide uptake, adenylate cyclase, and deoxyribonucleic acid synthesis in cultured rat thyroid cells J Clin Endocrinol Metab 1988 67 1 74 79 10.1210/jcem-67-1-74 3379138

[B4] Kraiem Z Sadeh O Blithe DL Nisula BC Human chorionic gonadotropin stimulates thyroid hormone secretion, iodide uptake, organification, and adenosine 3’,5’-monophosphate formation in cultured human thyrocytes J Clin Endocrinol Metab 1994 79 2 595 599 10.1210/jc.79.2.595 8045981

[B5] Carayon P Lefort G Nisula B Interaction of human chorionic gonadotropin and human luteinizing hormone with human thyroid membranes Endocrinology 1980 106 6 1907 1916 10.1210/endo-106-6-1907 6245856

[B6] Yoshimura M Hershman JM Pang XP Berg L Pekary AE Activation of the thyrotropin (TSH) receptor by human chorionic gonadotropin and luteinizing hormone in Chinese hamster ovary cells expressing functional human TSH receptors J Clin Endocrinol Metab 1993 77 4 1009 1013 10.1210/jc.77.4.1009 7691861

[B7] Norman RJ Dewailly D Legro RS Hickey TE Polycystic ovary syndrome Lancet 2007 370 9588 685 697 10.1016/S0140-6736(07)61345-2 17720020

[B8] Vanderpump MP Tunbridge WM French JM Appleton D Bates D Clark F Grimley Evans J Hasan DM Rodgers H Tunbridge F The incidence of thyroid disorders in the community: a twenty-year follow-up of the Whickham Survey Clin Endocrinol (Oxf) 1995 43 1 55 68 10.1111/j.1365-2265.1995.tb01894.x 7641412

[B9] Rajoria S Suriano R Shanmugam A Wilson YL Schantz SP Geliebter J Tiwari RK Metastatic phenotype is regulated by estrogen in thyroid cells Thyroid 2010 20 1 33 41 10.1089/thy.2009.0296 20067378 PMC2833180

[B10] Furlanetto TW Nguyen LQ Jameson JL Estradiol increases proliferation and down-regulates the sodium/iodide symporter gene in FRTL-5 cells Endocrinology 1999 140 12 5705 5711 10.1210/en.140.12.5705 10579335

[B11] Sosic-Jurjevic B Filipovic B Milosevic V Nestorovic N Manojlovic-Stojanoski M Brkic B Sekulic M Chronic estradiol exposure modulates thyroid structure and decreases T4 and T3 serum levels in middle-aged female rats Horm Res 2005 63 1 48 54 10.1159/000083139 15637454

[B12] Knudsen N Bulow I Laurberg P Perrild H Ovesen L Jorgensen T Low goitre prevalence among users of oral contraceptives in a population sample of 3712 women Clin Endocrinol (Oxf) 2002 57 1 71 76 10.1046/j.1365-2265.2002.01564.x 12100072

[B13] Arroyo A Laughlin GA Morales AJ Yen SS Inappropriate gonadotropin secretion in polycystic ovary syndrome: influence of adiposity J Clin Endocrinol Metab 1997 82 11 3728 3733 10.1210/jc.82.11.3728 9360532

[B14] Taylor AE McCourt B Martin KA Anderson EJ Adams JM Schoenfeld D Hall JE Determinants of abnormal gonadotropin secretion in clinically defined women with polycystic ovary syndrome J Clin Endocrinol Metab 1997 82 7 2248 2256 10.1210/jc.82.7.2248 9215302

[B15] Lewandowski KC Cajdler-Luba A Bienkiewicz M Lewinski A Women with oligo-/amenorrhoea and polycystic ovaries have identical responses to GnRH stimulation regardless of their androgen status: comparison of the Rotterdam and Androgen Excess Society diagnostic criteria Neuro Endocrinol Lett 2011 32 6 847 856 22286802

